# Cardiovascular β‐Adrenergic Receptor Distribution and Function: Influence of Species, Sex, Age, and Tissue

**DOI:** 10.1002/cph4.70159

**Published:** 2026-04-29

**Authors:** Basant Elsaid, Irakli Kopaliani, Birgit Zatschler, Ansam Seif, Stephan Speier, Andreas Deussen

**Affiliations:** ^1^ Institute of Physiology, Faculty of Medicine, Technische Universität Dresden Dresden Germany; ^2^ Department of Physiology Faculty of Medicine, Ain Shams University Cairo Egypt

**Keywords:** estrogen, menopause, preclinical models, translational research, vascular endothelium

## Abstract

Cardiovascular diseases remain the leading global cause of morbidity and mortality, with notable sex‐specific differences in prevalence and outcomes. Increasing evidence indicates that β‐adrenergic receptors (β‐AR) play a central role in cardiovascular regulation and that their expression, signaling, and responsiveness are modulated by estrogen. However, the precise mechanisms underlying β‐AR–estrogen interactions remain incompletely defined and highly context dependent. This mini‐review summarizes current knowledge of β‐AR subtypes, their cardiovascular distribution, and their signaling pathways, emphasizing how estrogen influences β‐AR function across species, sexes, ages, and cardiovascular tissues. Comparative analysis of data reveals substantial heterogeneity arising from tissue specificity, receptor subtype composition, and experimental models. These differences underscore the challenges of translating preclinical findings into human physiology. Future studies integrating multi‐species and sex‐specific approaches, including the development of genetic knockout models and validation using human vascular cells, are essential to bridge mechanistic insights with clinical relevance. Understanding the interplay between β‐AR signaling and estrogen is key to developing sex‐specific strategies for the prevention and treatment of cardiovascular diseases.

## Graphical abstract

Estrogen modulates β‐AR signaling in a context‐dependent manner influenced by species, sex, age, tissue, and vascular health, highlighting challenges in translating findings from experimental models to human cardiovascular physiology.

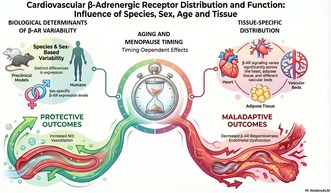

## 1 Introduction

Cardiovascular diseases (CVDs) continue to be a leading global cause of illness and death, with significant regional variations in mortality rates. Countries in Central and Eastern Europe, as well as Central Asia, report the highest CVD‐related deaths worldwide, though mortality rates among women are generally lower than those in men (WHO 2024). In the United States, CVD remains the most prevalent cause of death, accounting for approximately 702,880 fatalities in 2022, with coronary artery disease affecting an estimated 5% of adults aged 20 and older (CDC 2024). Meanwhile, CVD mortality in the Middle East and North Africa has almost doubled between 1990 and 2019. The burden of CVD is particularly pronounced in countries with high Disability‐Adjusted Life Years (DALYs), including Afghanistan, Egypt, and Yemen (Alhuneafat et al. 2024).

There is growing recognition that sex‐specific differences influence the prevalence, mechanisms, and treatment outcomes of CVDs (Regitz‐Zagrosek and Gebhard 2023). Conditions such as hypertension (HTN) and ischemic heart disease (IHD) do not manifest identically in men and women, with distinct variations in risk factors, disease progression, and therapeutic responses. HTN remains one of the most significant modifiable risk factors, contributing to approximately 10.8 million deaths worldwide in 2019 (Aryan et al. 2020). The role of the sympathetic nervous system (SNS) in regulating blood pressure is well established, and notable sex differences in SNS activity have been reported (Corbi et al. 2024). In younger women there is increased responsiveness of vascular β‐ARs compared to men, which is critical for counteracting the effects of α‐AR‐mediated vasoconstriction (Liccardo et al. 2022; Kopaliani et al. 2024). Beyond the release of neurotransmitters through the SNS, catecholamines like epinephrine and norepinephrine (NE) are also secreted by the adrenal medulla. These adrenal‐derived catecholamines interact with adrenergic receptors and play a pivotal role in cardiovascular regulation and other physiological processes (Slotkin 1986).

As women progress from the premenopausal to the postmenopausal stage, notable changes in vascular tone regulation occur (Liccardo et al. 2022). Aging in women is associated with increased SNS activity, leading to sustained hemodynamic stress that promotes adverse cardiovascular remodeling. Studies demonstrate that postmenopausal women experience greater blood pressure increases during stress compared to premenopausal women or those receiving hormone replacement therapy (HRT). Similarly, vasoconstrictive responses to NE are more pronounced after menopause, whereas HRT attenuates these effects, restoring vascular responsiveness to levels similar to those in younger women (Corbi et al. 2024). The cardiovascular benefits of HRT in postmenopausal women are partly attributed to its ability to rebalance SNS activity and normalize β‐AR function (Liccardo et al. 2022).

The interplay between β‐ARs and estrogen is increasingly recognized as a critical factor in sex‐based differences in cardiovascular regulation. Estrogen has been shown to modulate β‐AR expression and function in vascular tissues while also influencing SNS‐mediated catecholamine release. These mechanisms collectively contribute to differences in blood pressure regulation between men and women (Corbi et al. 2024). Given that sex differences in adrenergic signaling may impact therapeutic outcomes, exploring these mechanisms further could lead to improved strategies for managing HTN and other CVDs (Machuki et al. 2018). Current CVD management guidelines are primarily based on clinical trials in which women have been historically underrepresented (Garcia et al. 2016). Recently, greater emphasis has been placed on developing recommendations that account for developing guidelines that incorporate patients' age and sex (ESC 2021).

Despite growing evidence that estrogen modulates β‐AR signaling, the precise mechanisms underlying this interaction remain poorly defined. Current research is highly heterogeneous, encompassing studies performed in different vascular beds, species, sexes, and experimental conditions, each yielding variable and sometimes conflicting outcomes. Such methodological and biological diversity makes it challenging to determine which findings are truly translational to human physiology. Collectively, these gaps impede a unified understanding of how estrogen shapes β‐AR–mediated regulation of the cardiovascular system and how these mechanisms contribute to sex‐specific disease risk and therapeutic response. The aim of this mini‐review is to summarize current knowledge on β‐AR subtypes, their vascular distribution, and signaling mechanisms, with a particular focus on how estrogen influences β‐AR functions across sexes and species. By integrating findings from molecular, physiological, and comparative studies, this mini‐review seeks to clarify sex‐specific differences in β‐AR signaling within the cardiovascular system and highlight their potential implications for translational research and therapeutic strategies in cardiovascular disease.

## 2 Methods

This mini‐review was conducted through a targeted literature search in PubMed using combinations of the following keywords: β‐AR, estrogen, cross talk, ovariectomy, sex differences, cardiovascular system, cardiovascular disease, rat, mouse, animals, human, cells, endothelium, arteries, gene expression, protein expression, timing/temporal hypothesis, aging, menopause, endothelial dysfunction, and translational research. Articles published in English between 2005 and 2025 were considered. Preference was given to experimental and clinical studies that directly examined the interaction between β‐AR signaling and estrogen or sex hormones in the context of cardiovascular regulation. Reference lists of relevant articles and recent reviews were also screened to identify additional sources. Both animal and human studies were included to highlight species‐specific findings and translational implications.

### 2.1 Overview of β‐AR Receptor Subtypes and Signaling

β‐ARs are key mediators of SNS activity, activated primarily by catecholamines such as epinephrine and NE (do Vale et al. 2019). Since their discovery in the early 20th century, research on β‐ARs has greatly advanced, facilitating significant progress in cardiovascular therapeutics, including the development of β‐AR‐specific blockers, some of which possess intrinsic sympathomimetic activity (ISA) (Fumagalli et al. 2020).

Structurally, β‐ARs consist of 408 to 477 amino acids, characterized by extended C‐terminal regions and abbreviated third intracellular loops, both of which are essential for receptor regulation and function. Three distinct genes encode the principal β‐AR subtypes (β1, β2, and β3). Notably, the β1‐ and β2‐AR genes lack introns, resulting in a single receptor form (Bylund 2009). The β3‐AR gene shows notable differences across species. In humans, dogs, and monkeys, it generally consists of two exons and one intron, producing isoforms with minor C‐terminal variations. In rodents, the gene contains three exons and two introns, and alternative splicing generates multiple isoforms that differ in their C‐terminal sequences and tissue‐specific expression. Despite these differences, the encoded proteins share the characteristic seven‐transmembrane G protein‐coupled receptor (GPCR) structure, with the highest sequence conservation in the transmembrane domains and greater divergence in the C‐terminus and intracellular loops (Dessy and Balligand 2010). A potential fourth β‐AR subtype, β4, has been proposed, though its existence in the cardiovascular system remains under investigation (Liccardo et al. 2022).

Functionally, β‐ARs mediate cardiovascular effects via interaction with heterotrimeric G proteins composed of Gα, Gβ, and Gγ subunits. Upon ligand binding, β‐ARs predominantly couple to Gs proteins, activating adenylyl cyclase (AC) and elevating cyclic adenosine monophosphate (cAMP) levels. Although β1‐, β2‐, and β3‐ARs mainly stimulate Gs proteins, β2‐ and β3‐ARs can also engage Gi proteins, introducing additional regulatory complexity (Liccardo et al. 2022).

β_1_‐ and β_2_‐ARs predominantly couple to Gs proteins, stimulating AC, increasing cAMP production, and enhancing cardiac contractility (Bristow et al. 1990; Liccardo et al. 2022). In addition to this classical pathway, β_2_‐AR can also couple to Gi proteins, particularly in rat and mouse cardiomyocytes (Xiao et al. 1995; Xiao et al. 1999; Xiao 2001; Liccardo et al. 2022). This pathway serves as a regulatory mechanism that limits excessive stimulation and provides cardioprotection; as β2‐ARs can switch to Gi coupling under high adrenaline concentrations, a process known as “stimulus‐mediated trafficking” (Liccardo et al. 2022). In contrast, β_3_‐AR exhibit distinct, species‐ and tissue‐dependent signaling. In the human ventricle, β_3_‐ARs are predominantly coupled to Gi proteins; however, rather than inhibiting AC, they primarily activate the nitric oxide (NO) pathway, likely through endothelial nitric oxide synthase (eNOS), leading to cGMP production and negative inotropic effects (Gauthier et al. 1996; Gauthier et al. 1998; Cannavo and Koch 2017). A similar Gi predominance has been reported in rat myocardium (Gauthier et al. 1996; Zhang et al. 2005; Cannavo and Koch 2017). In mice, β_3_‐AR signaling is isoform‐specific, with β_3_a‐AR preferentially coupling to Gs, whereas β_3_b‐AR can interact with both Gs and Gi (Hutchinson et al. 2002; Sato et al. 2005). In adipose tissue, β_3_‐AR coupling also varies by species: it is mainly Gs‐mediated in human adipocytes (Cannavo and Koch 2017), while in mouse and rat it involves both Gs and Gi pathways (Chaudhry et al. 1994; Bégin‐Heick 1995). In vascular endothelium, β_3_‐AR signaling is generally associated with Gi coupling and NO‐mediated vasodilation (Rozec and Gauthier 2006).

**FIGURE 1:**
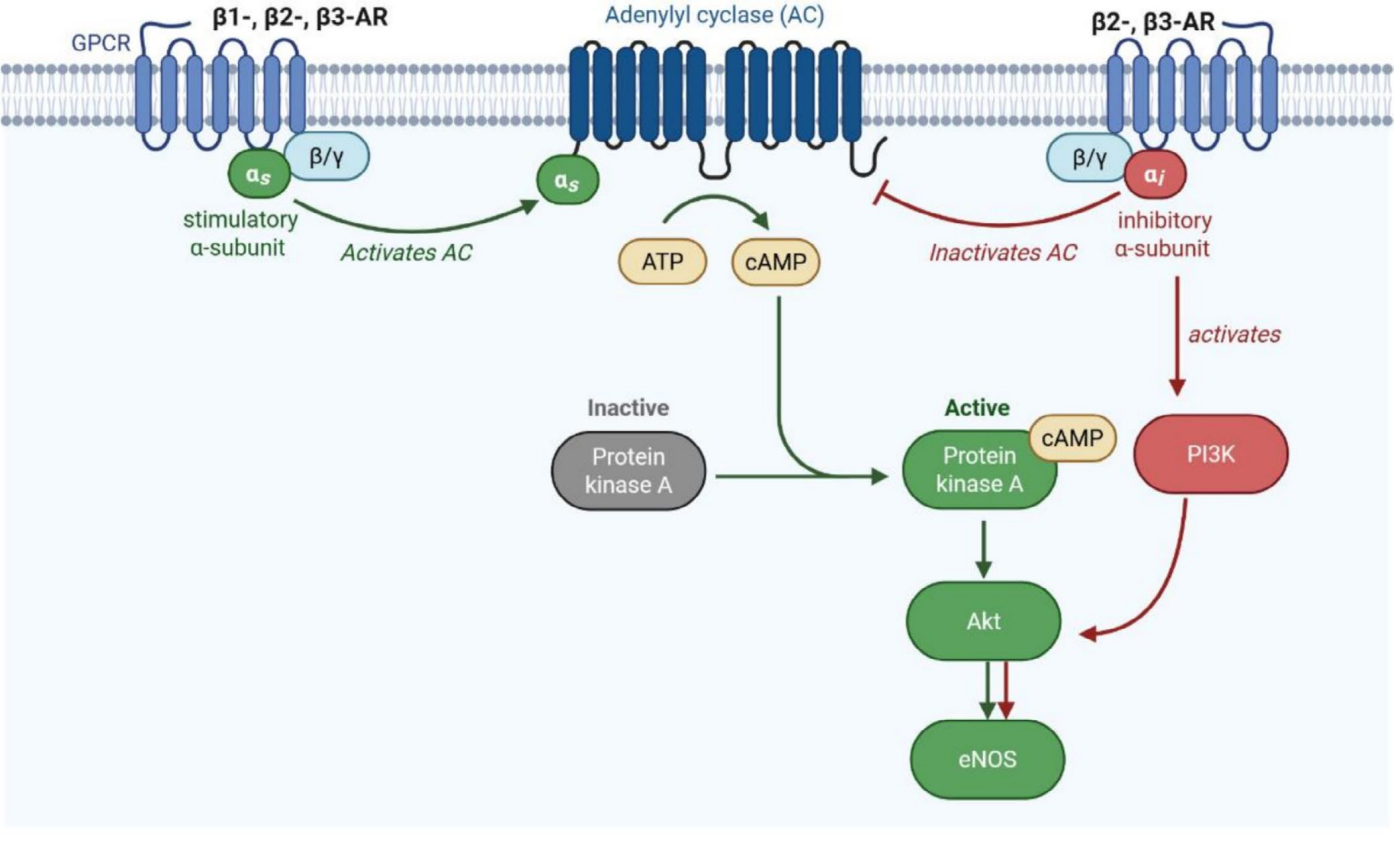
Mechanistic overview of β‐AR receptor subtypes in vascular system. This figure depicts the G protein‐coupled signaling pathways of different β‐AR subtypes. Created with BioRender.com.

Feedback regulation of β1‐ & β2‐ARs occurs via protein kinase A (PKA)‐dependent phosphorylation, which diminishes their coupling to Gs proteins, promoting receptor desensitization and reducing cAMP production. Furthermore, PKA‐mediated phosphorylation of β2‐ARs facilitates coupling to Gi proteins, leading to the activation of alternate pathways such as Gβγ/PI3K/Akt signaling (Oliver et al. 2019). In contrast, β3‐ARs are relatively resistant to desensitization due to the absence of consensus PKA and G protein–coupled receptor kinase (GRK2) phosphorylation sites in their cytoplasmic C‐terminal regions. Consequently, β3‐ARs maintain signaling via the eNOS/NO/cGMP/PKG axis even during prolonged stimulation (Machuki et al. 2018). Nevertheless, emerging evidence suggests that GRK2 may transiently desensitize β3‐ARs through phosphorylation‐independent mechanisms, highlighting alternative pathways of receptor regulation (Corbi et al. 2024). The signaling mechanisms of β‐AR receptor (β‐AR) subtypes in the vascular system are illustrated in Figure 1.

Under non‐selective β‐AR stimulation, activation of AC elevates intracellular cAMP levels, leading to PKA activation and phosphorylation of downstream targets such as Akt and eNOS. The resulting increase in NO production promotes vasodilation through activation of soluble guanylyl cyclase (sGC) and cyclic guanosine monophosphate (cGMP) generation in vascular smooth muscle cells (VSMCs) (Liccardo et al. 2022). Evidence from male Wistar rat pulmonary arteries supports this canonical pathway, where prolonged isoprenaline exposure enhances eNOS‐derived NO release and thus maintains vasodilatory tone (Davel et al. 2015). However, sustained β‐AR stimulation with isoproterenol in male C57BL/6 mice aorta can disrupt this balance, leading to eNOS uncoupling, reduced NO bioavailability, and increased oxidative stress (Bernak‐Oliveira et al. 2021). This effect results from increased activation of β_2_‐AR/Giα signaling pathway and ERK1/2 phosphorylation at Thr_202_/Tyr_204_ (Davel et al. 2014). Under such conditions, compensatory activation of neuronal nitric oxide synthase (nNOS), along with hydrogen peroxide (H_2_O_2_) and caveolin‐1, a complex that is critical for processes such as eNOS activation, partially preserves endothelial relaxation (Bernak‐Oliveira et al. 2021).

Additional mechanisms, including cyclooxygenase (COX) activity, further modulate β‐AR‐mediated vasodilation. In humans, COX inhibition enhances non–subtype‐specific β‐AR vasodilation in young obese adults and healthy young men, suggesting that COX‐derived factors can suppress NO synthase activity and attenuate β‐AR function (Limberg et al. 2016; Fujii et al. 2017). Furthermore, in humans, Nebivolol has been shown to enhance the contribution of both NO and endothelial‐derived hyperpolarizing factors (EDHFs) to basal vasomotor tone in the forearm vessels of hypertensive African American individuals (Neuman et al. 2016). Complementary studies in human umbilical vein endothelial cells (HUVECs) demonstrated that Nebivolol‐induced NO release occurs with β_3_‐AR stimulation exclusively through its active metabolites (Mason et al. 2013). However, in mice thoracic aorta segments, the NO‐dependent vasodilatory effect of Nebivolol has been shown to occur via β_2_‐AR activation rather than β_3_‐AR stimulation (Broeders et al. 2000). Overall, these previous findings indicate that β‐AR–mediated NO‐dependent vasodilation is species‐ and vascular bed–dependent.

### 2.2 Species‐, Vascular Bed‐, Endothelium, and Sex‐Dependent Variability in β‐AR Receptor Expression and Function

Systematic studies on β‐AR expression across vascular beds and species remain limited. Available data indicate that all three β‐AR subtypes (β_1_, β_2_, and β_3_) are present in the aorta and mesenteric arteries of Wistar rats, Wistar Kyoto rats (WKY), and spontaneously hypertensive rats (SHRs); in the cremaster arteries of male Sprague–Dawley rats; and in the internal mammary arteries of humans (Flacco et al. 2013; Al‐Gburi et al. 2017a; Riedel et al. 2019; Saunders et al. 2021). In male Wistar rats, β_3_‐AR expression is lowest in mesenteric arteries and highest in the aorta (Flacco et al. 2013). β_1_‐ and β_2_‐ARs are detectable in the pulmonary vessels of male C57BL6 mice (Leblais et al. 2008). β_3_‐ARs are expressed in multiple vascular beds across species, including the renal arterioles of male Sprague–Dawley rats, the large pulmonary arteries and aorta of male Wistar rats, and the coronary arterioles of humans with ischemic heart disease (Rautureau et al. 2002; Dessy et al. 2004; Feng et al. 2012; Davel et al. 2015).

**FIGURE 2:**
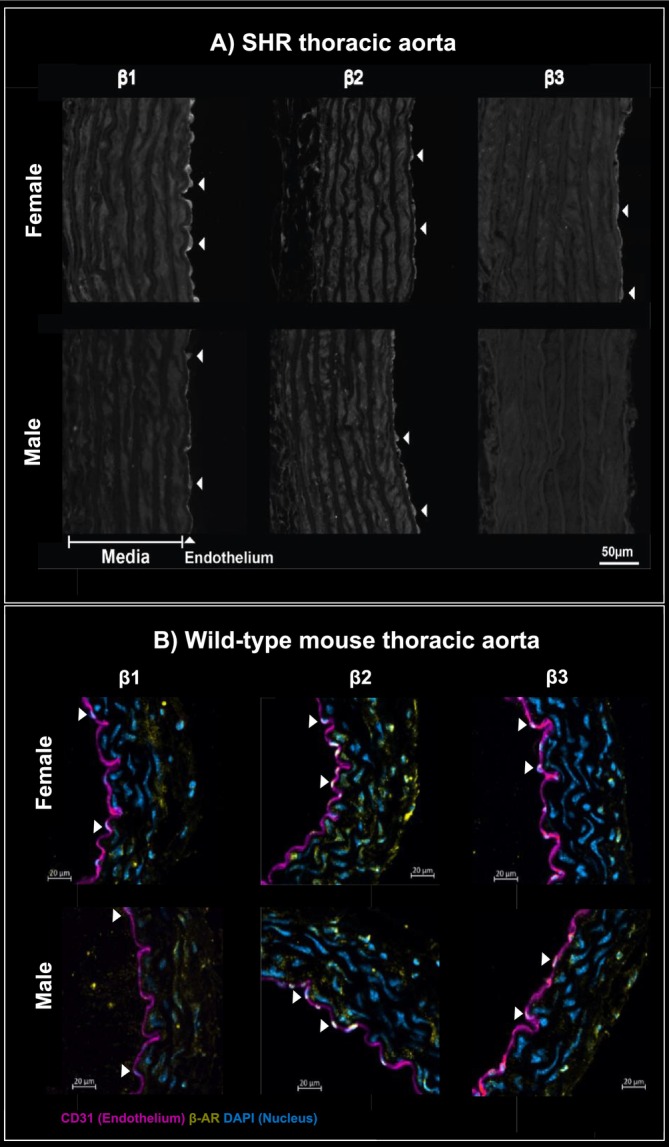
β‐AR localization in the thoracic aorta of female and male SHRs, and wild‐type C57BL6 mice. Immunofluorescence images correspond to β_1_‐AR, β_2_‐AR, and β_3_‐AR expression in female and male, respectively, in 12‐week‐old SHRs (panel A) and 16–18‐week‐old wild‐type C57BL6 mice (panel B). In panel (B), endothelial cells are labeled with CD31 and Alexa Fluor 647 (magenta), nuclei are stained with DAPI (blue), and β‐ARs are detected using subtype‐specific antibodies conjugated to Alexa Fluor 555 (yellow). The single‐channel fluorescence and control images corresponding to the merged IF images are shown in Figures A1 and A2, respectively. Panel (A) is created based on data from Al‐Gburi et al. (2017a).

Rat and mouse models are the most frequently used in preclinical cardiovascular research; however, with respect to sex‐specific differences in β‐AR expression, these species exhibit notable divergence. In 12‐week‐old SHRs, β_1_‐ARs show greater endothelial expression in females than in males' thoracic aorta, whereas β_2_‐AR expression is similar between sexes. In contrast, β_3_‐ARs are absent in the male endothelium (Al‐Gburi et al. 2017a). In contrast, in 16–18‐week‐old wild‐type C57BL6 mice, no sex differences were observed for any β‐AR subtype, with β_1_‐, β_2_‐, and β_3_‐AR localization being equivalent in males and females (Figure 2).

The contribution of individual β‐AR subtypes to vasodilation varies markedly with vascular bed, species, strain, sex, and endothelial involvement. β_1_‐, β_2_‐, and β_3_‐ARs play a key role in retinal arterioles of male Wistar rats, in the aorta and mesenteric arteries of WKY rats, and SHRs, in the cremaster arteries of male Sprague–Dawley rats, and in the internal mammary arteries of humans (Flacco et al. 2013; Al‐Gburi et al. 2017a; Mori et al. 2017; Riedel et al. 2019; Saunders et al. 2021). β_1_‐ and β_2_‐ARs are predominant in the coronaries of Sprague–Dawley rats, in the large pulmonary arteries, common carotid, and aorta of Wistar rats, and in pulmonary vessels of male C57BL6 mice (O'Donnell and Wanstall 1981; Abdelrahman et al. 1990; Satake et al. 1997; Chiba and Tsukada 2001; Leblais et al. 2008; Davel et al. 2015). In the male human forearm vasculature, vasodilatory responses to β‐AR agonists are predominantly mediated by β_2_‐ARs (Dawes et al. 1997). Conversely, β_1_‐ARs play a key role in vasodilation in human coronary vessels and in several animal species, including bovine, monkey, and dog coronary vessels (Toda and Okamura 1990; Purdy et al. 1988). β_3_‐ARs contribute significantly to vasodilation in multiple vascular beds across species, including the renal arterioles of male Sprague–Dawley rats, the large pulmonary arteries of male Wistar rats, the coronary arterioles of humans with ischemic heart disease, and the chicken basilar artery (Dessy et al. 2004; Feng et al. 2012; Davel et al. 2015; Wu et al. 2023).

Endothelium‐dependent β‐AR mediated responses have been reported across multiple species and vascular beds, although subtype involvement varies. In Sprague–Dawley rat cremaster arteries, all three β‐AR subtypes (β_1_, β_2_, and β_3_) exhibit endothelium‐dependent effects (Saunders et al. 2021). In contrast, β_1_‐ and β_2_‐AR–mediated responses are endothelium‐dependent in Wistar rat large pulmonary arteries, as well as in aorta, C57BL6 mouse pulmonary arteries, and human forearm vasculature (Dawes et al. 1997; Satake et al. 1997; Leblais et al. 2008; Davel et al. 2015). β_2_‐ and β_3_‐AR endothelium‐dependent effects have been demonstrated in Wistar rat aorta (Flacco et al. 2013). β_3_‐AR–mediated responses are endothelium‐dependent in Wistar rat aorta, Sprague–Dawley rat renal arterioles, chicken basilar artery, and human coronary arterioles from cardiac surgery patients (Rautureau et al. 2002; Dessy et al. 2004; Feng et al. 2012; Wu et al. 2023). β_3_‐AR–mediated endothelium‐dependent vasodilation in the male Sprague–Dawley rat cremaster arteries become apparent only after β_1_‐ and β_2_‐AR blockade, suggesting that constitutive activity of these subtypes may suppress β_3_‐AR function in resistance arteries (Saunders et al. 2021).

Sex differences in vascular responses to β‐AR agonists have been demonstrated in both rats and humans. Studies from our research group revealed that vasorelaxation to β_1_/β_2_‐AR agonist isoprenaline and β_3_‐AR agonist BRL37344 is significantly greater in females than in males (Al‐Gburi et al. 2017a; Riedel et al. 2019). These differences were observed in the thoracic aorta of SHRs, and were confirmed in normotensive WKY rats, Wistar rats, and the internal mammary arteries of humans (Al‐Gburi et al. 2017a; Riedel et al. 2019). Similar findings were observed in Wistar rat mesenteric arteries (Riedel et al. 2019). Preconstriction with either KCl or phenylephrine did not alter the sex difference, indicating that it is independent of the preconstrictor used (Riedel et al. 2019).

**FIGURE 3:**
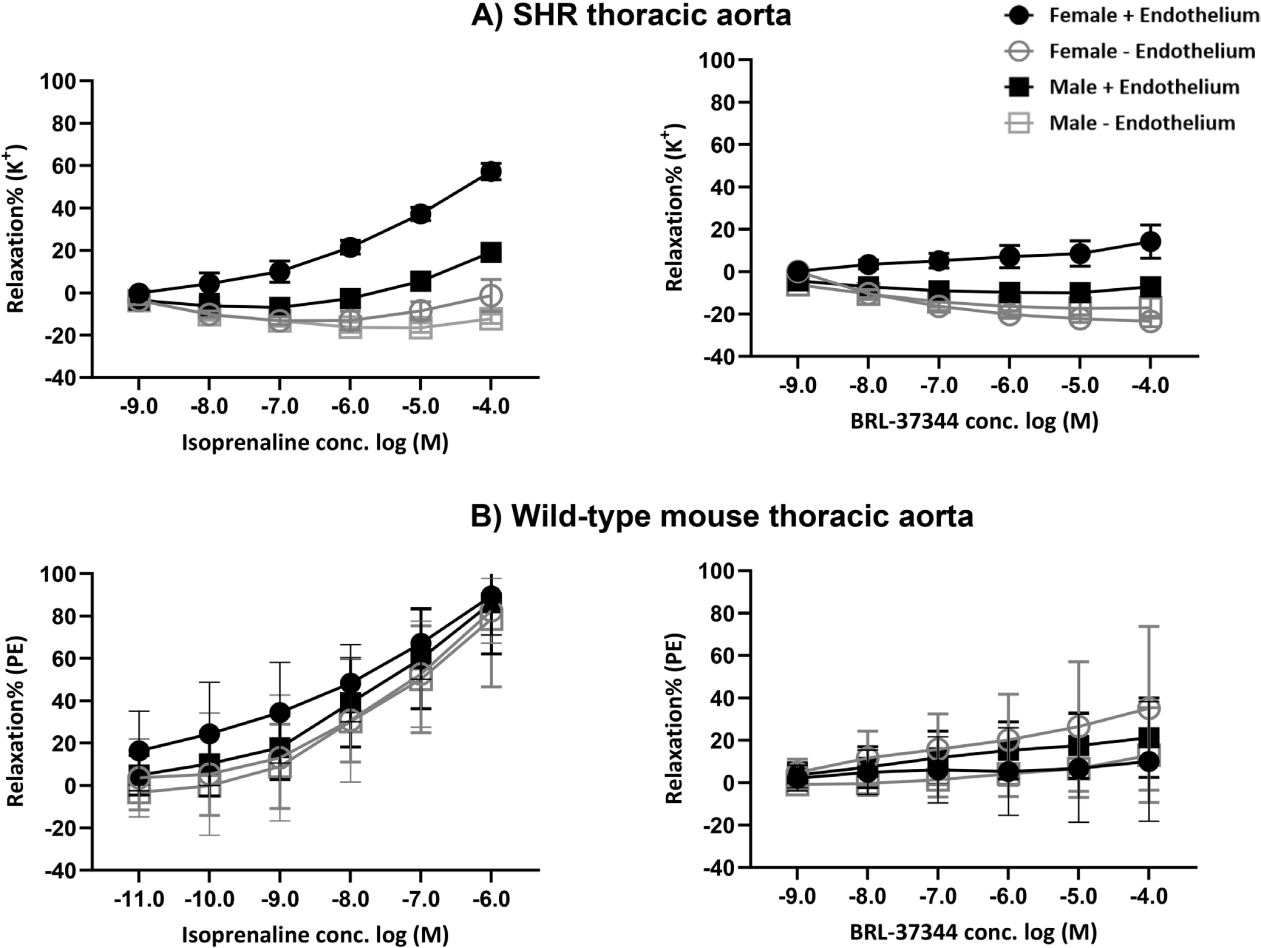
Vasorelaxant effects of β‐AR agonists in thoracic aorta from SHRs and wild‐type C57BL6 mice. The graphs illustrate the vasorelaxant responses of female and male aortic rings, with and without endothelium, to β‐AR agonists. Panel (A) shows the effects of the β_1_/β_2_‐AR agonist isoprenaline and the β_3_‐AR agonist BRL‐37344 in female and male SHRs. Significant sex differences are observed in SHRs, and these differences are endothelium‐dependent, as removal of the endothelium abolishes the enhanced responses in females. Panel (B) depicts the effects of the same β‐AR agonists in female and male wild‐type mice, where no significant sex differences are detected, and endothelial removal has no measurable effect. Experiments were performed with *n* = 6 for both SHRs and wild‐type mice. All values are presented as mean ± SD. Panel (A) is created based on data from Al‐Gburi et al. (2017a).

The sex differences were abolished by endothelial removal, demonstrating that enhanced vasorelaxation in females is endothelium‐dependent and likely driven by higher endothelial expression of β_1_‐ and β_3_‐ARs. mRNA levels of β_1_‐ and β_3_‐ARs were significantly higher in female aortas compared to males across SHRs, WKY rats, Wistar rats, and human internal mammary arteries, whereas β_2_‐AR expression remained similar between sexes (Al‐Gburi et al. 2017a; Riedel et al. 2019). In contrast, in mice, no significant sex differences in vasorelaxation to the same β‐AR agonists were observed, and endothelial integrity had no measurable impact on these responses (Figure 3). This highlights a key species difference, as endothelial β‐AR expression underlies sex‐dependent responses in rats, whereas the mouse vasculature does not exhibit this pattern.

These species‐, sex‐, vascular bed‐specific differences in β‐AR expression, and endothelial role summarized in Table 1, underscore the importance of considering model‐specific variation when interpreting experimental results and extrapolating to human physiology.

**TABLE 1:** β‐AR expression across vascular beds, species, sex, and endothelial role.

Species (strain, age)	Sex	Vascular bed	Method	β_1_‐AR	β_2_‐AR	β_3_‐AR	Endothelium role	References
Rat (Wistar, 12 w)	M	Large pulmonary artery	In vivo (pharmacological)	+	+	NA	Dependent	Davel et al. (2015)
Rat (Wistar, 5–8 w)	M	Pulmonary artery	In vitro (pharmacological)	+	+	NA	NA	O'Donnell and Wanstall (1981)
Rat (Wistar, 7–10 w)	M	Retinal arterioles	In vivo (pharmacological)	+	+	+	NA	Mori et al. (2017)
Rat (Wistar, age NA)	M	Aorta	In vitro (pharmacological)	+	+	NA	Dependent	Satake et al. (1997)
Rat (Wistar, 6–8 w)	M/F	Common carotid artery	In vitro (pharmacological)	+ (sex differences NA)	++ (> β_1_AR) (Sex differences NA)	NA	Independent	Chiba and Tsukada (2001)
Rat (Wistar, age NA)	M	Aorta	In vitro (mRNA, IHC)	NA	NA	+	Dependent	Rautureau et al. (2002)
Rat (Wistar, age NA)	M	Aorta	In vitro (pharmacological, mRNA, IF)	+	+	++ (highest, mostly elastic lamina)	β_2_/β_3_ dependent	Flacco et al. (2013)
Rat (Wistar, age NA)	M	Mesenteric artery	In vitro (pharmacological, mRNA, IF)	++	++	+ (lowest)	Independent	Flacco et al. (2013)
Rat (SHR/WKY, 12 w)	M/F	Aorta	In vitro (pharmacological, mRNA, IF)	+ (↑ F > M)	+ (F = M)	+ (↑ F > M)	Dependent	Al‐Gburi et al. (2017a)
Rat (Wistar, 12 w)	M/F	Aorta, mesenteric artery	In vitro (pharmacological, mRNA)	+ (↑ F > M)	+ (F = M)	+ (↑ F > M)	Dependent	Riedel et al. (2019)
Rat (Sprague–Dawley, age NA)	M	Renal arterioles	In vitro (pharmacological, IHC)	NA	NA	+	Dependent	Feng et al. (2012)
Rat (Sprague–Dawley, age NA)	M	Coronary, skeletal muscle artery	In vivo (pharmacological)	+	+	NA	NA	Abdelrahman et al. (1990)
Rat (Sprague–Dawley, 6 w)	M	Cremaster artery	In vitro (pharmacological, mRNA, IF)	+	+	+ (apparent after β_1_/β_2_ blockade)	Dependent	Saunders et al. (2021)
Mouse (C57BL6, 11–14 w)	M	Pulmonary artery	In vitro (pharmacological, IHC)	+	+	−	Dependent	Leblais et al. (2008)
Mouse (C57BL6, 16–18 w)	M/F	Aorta	In vitro (pharmacological, IF)	+ (no sex differences)	+ (no sex differences)	+ (no sex differences)	Independent	Figures 2 and 3
Chicken (age NA)	NA	Basilar artery	In vitro (pharmacological)	−	−	+	Dependent	Wu et al. (2023)
Bovine (age NA)	NA	Coronary artery	In vitro (pharmacological)	++	+	NA	NA	Purdy et al. (1988)
Human/Monkey/Dog (age NA)	NA	Coronary, renal, mesenteric artery	In vitro (pharmacological)	++	+	NA	NA	Toda and Okamura (1990)
Human (healthy, 18–30 y)	M	Forearm vasculature	In vivo (pharmacological)	+	++	NA	Dependent	Dawes et al. (1997)
Human (cardiac surgery patients, age NA)	NA	Coronary arterioles	In vitro (pharmacological, mRNA, protein, IHC)	−	−	+	Dependent	Dessy et al. (2004)
Human (45–85 y; 48–55y)	M/F	Internal mammary artery	In vitro (pharmacological, mRNA)	+ (↑ F > M)	+ (F = M)	+ (↑ F > M)	Dependent	Al‐Gburi et al. (2017a) and Riedel et al. (2019)

*Note:* w, weeks; M, male; F, female; NA, not assessed; +, detectable; ++, comparatively higher; −, not detectable; Dependent/Independent, endothelium‐dependent or ‐independent effect; ↑ F > M, higher in females than males; F = M, no sex difference. The “Method” column indicates both the experimental setting and the type of analysis performed. “In vivo” refers to studies conducted in intact organisms, whereas “in vitro” refers to studies in isolated tissues or cells. Pharmacological approaches assess functional vascular responses to receptor stimulation or inhibition, while mRNA, protein, immunohistochemistry (IHC), and immunofluorescence (IF) indicate receptor expression at the molecular or protein level.

### 2.3 Interplay Between Estrogen and β‐AR Receptor Signaling in the Cardiovascular System

Systemic regulation of catecholamine homeostasis, including the inhibitory effects of estrogen on catecholamine secretion, has been extensively studied in rats, particularly in the adrenal gland. These studies highlight estrogen's critical role in modulating sympathetic output at the systemic level in this species. For example, gonadectomized female rats exhibit elevated catecholamine levels compared to males, a dysregulation that is restored by estrogen supplementation (Gomes et al. 2012). Furthermore, estrogen has been shown to directly inhibit catecholamine release from adrenal chromaffin cells, emphasizing its broad regulatory influence on sympathetic neurotransmitter secretion (Sudhir et al. 1997; Sung et al. 1999).

Estrogen can influence β‐ARs both through direct modulation of receptor activity and via downstream signaling pathways. A central component of this regulation is the localization of estrogen receptors (ERs) and β‐ARs within specialized membrane microdomains called caveolae, which facilitate specific receptor interactions and signaling. For example, in endothelial cells, β_3_‐ARs in Chinese hamster ovary (CHO‐K1) cells (Sato et al. 2012), ERα in immortalized ovine pulmonary artery endothelial cells (iPAECs) (Chambliss et al. 2000) and MCF‐7 cells (Schlegel et al. 1999), and ERβ in primary ovine endothelial cells from intrapulmonary arteries of fetal lambs (Chambliss et al. 2002) all associate with caveolin‐1, a key interaction for eNOS activation and vascular relaxation. In Wistar rat cardiomyocytes, β_1_‐AR and β_2_‐AR are primarily localized to caveolin‐3 (Rybin et al. 2000). Notably, the Gαs/AC/cAMP pathway and the Gαi/PI3K/Akt pathway often exert opposing effects; elevated cAMP levels can inhibit Akt kinase activity, illustrating a dynamic antagonism between these signaling cascades (Machuki et al. 2018). Such interactions are likely important for maintaining vascular homeostasis and highlight the intricate cross‐talk between β‐ARs and ERs.

The signaling mechanisms of β‐ARs and ERs are closely interconnected through the GPCR system, involving both Gαs and Gαi proteins. β_1_‐ARs primarily couple with Gαs, whereas β_2_‐ARs, β_3_‐ARs, ERα, ERβ, and the G‐protein‐coupled estrogen receptor (GPR30, GPER) can interact with both Gαs and Gαi subunits. Gαs activation stimulates AC, increasing intracellular cAMP levels, which subsequently activate downstream effectors such as PKA and exchange protein directly activated by cAMP (EPAC) (Machuki et al. 2018). For example, in porcine coronary arteries, GPR30 activation initiates a signaling cascade that elevates PKA and EPAC activity, mirroring the effects of β_1_‐ and β_2_‐AR stimulation (Thomas et al. 2005; Yu et al. 2014; Zucchetti et al. 2014).

**FIGURE 4:**
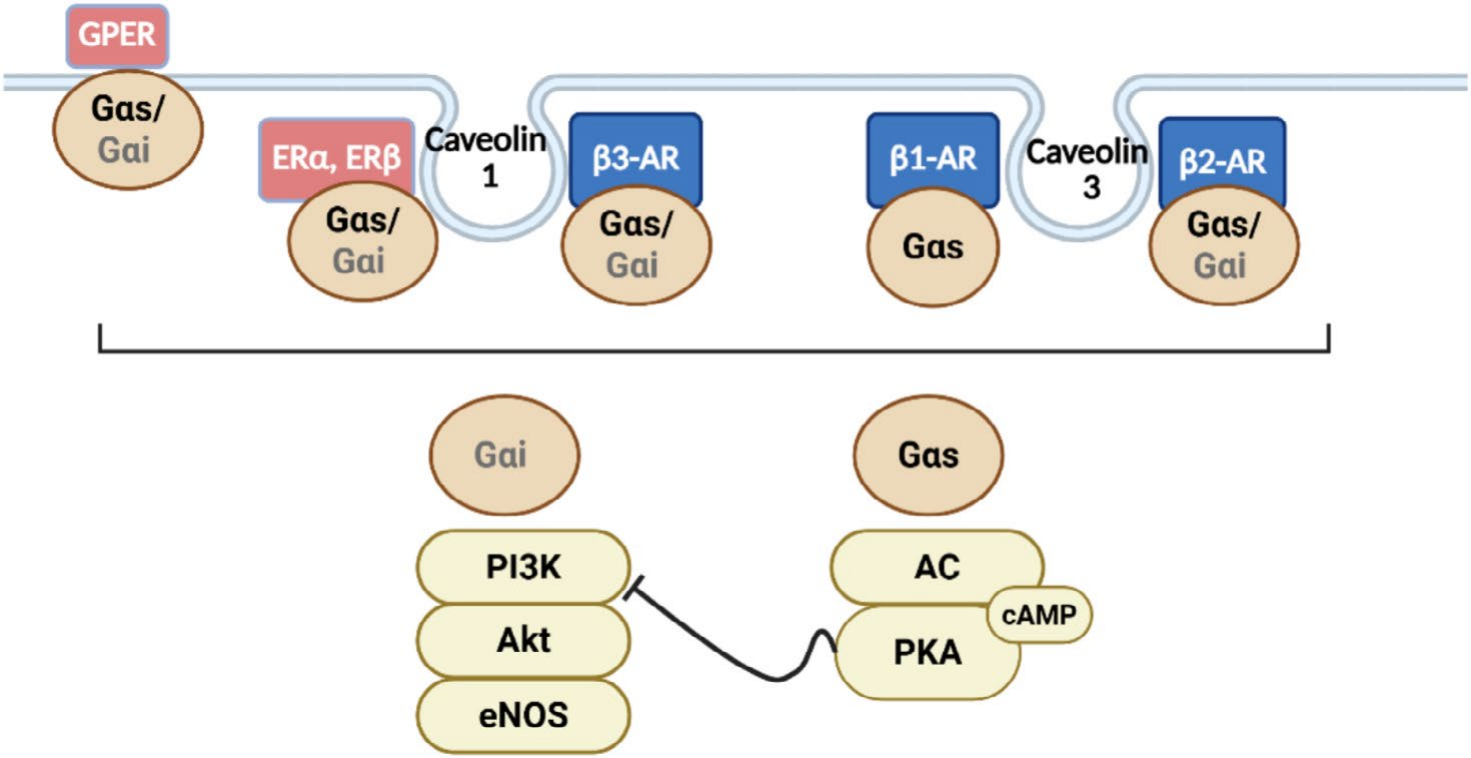
Cross‐talk between ERs and β‐ARs. Schematic representation of the interactions between membrane‐bound ERs and β‐ARs within caveolae, illustrating the Gαs‐ and Gαi‐mediated activation of the cAMP/PKA and PI3K/Akt signaling pathways. Created with BioRender.com.

Conversely, the GPCR/Gαi/PI3K/Akt pathway, involving ERα, ERβ, and GPR30, parallels β_2_‐ and β_3_‐AR coupling to Gαi (Machuki et al. 2018). ERα also enhances PKA activity in vascular smooth muscle cells (VSMCs) of the rat aorta, highlighting estrogen's modulation of Gαs/cAMP signaling (Ding et al. 2009). Notably, the Gαs/AC/cAMP and Gαi/PI3K/Akt pathways often exert opposing effects, such as cAMP‐mediated inhibition of Akt, reflecting a dynamic antagonism that fine‐tunes cardiovascular signaling (Machuki et al. 2018). Overall, β‐AR and ER signaling in cardiac and vascular cells is highly integrated, with Gαs and Gαi pathways coordinating complex cellular responses. This interplay is schematically depicted in Figure 4, showing their coordinated localization in caveolae and the engagement of both Gαs‐ and Gαi‐mediated pathways, ultimately activating PKA‐dependent and PI3K/Akt signaling routes.

Structurally, GPR30 shares notable similarities with β‐ARs, enabling functional interactions between these receptor systems. GPR30 contains phosphorylation sites for PKA and PDZ domain–binding motifs, which facilitate its association with A‐kinase anchoring protein 5 (AKAP5) and integration into β‐AR–related signaling pathways. Emerging evidence suggests that GPR30 can inhibit AC activity through its association with the membrane‐associated guanylate kinase (MAGUK)/AKAP5 complex, thereby modulating cAMP production in the context of β‐AR stimulation (Broselid et al. 2014). Additionally, GPR30 may influence cAMP degradation via phosphodiesterase (PDE) regulation, a mechanism also mediated by AKAP5 and analogous to processes observed in β‐AR signaling (Machuki et al. 2018).

### 2.4 Estrogenic Modulation of β‐AR Receptor Expression and Function

The modulatory effects of estrogen on vascular β‐AR signaling have been investigated using pharmacological approaches. Nebivolol, for instance, promotes vasodilation via ER–dependent NO signaling, particularly through the β_3_‐AR/ERβ/NO pathway. These mechanisms have been studied in rats, focusing on endothelial cells in the renal afferent arterioles and aorta (Garbán et al. 2004; Feng et al. 2012). Additionally, selective blockade of β_1_‐AR (CGP20712A) and β_2_‐AR (ICI118551) abolishes the cardioprotective effects of G‐1, a selective GPER agonist, in a heart failure model of female Sprague–Dawley rats. Treatment with G‐1 normalized the expression of β_1_‐AR and increased the expression of β_2_‐AR and improved cardiac function (Kang et al. 2012).

Studies from our group in female Wistar rats demonstrated that ovariectomy (OVX) reduces β_1_‐ and β_3_‐AR expression in the thoracic aorta, effects that were restored by estrogen replacement but not by progesterone (Riedel et al. 2019). Conversely, other studies reported increased β_2_‐AR mRNA levels in the mesenteric arteries of estrogen‐treated rats compared to placebo‐treated controls (Tazumi et al. 2018). Similarly, in younger women, β_2_‐AR stimulation produces greater increases in blood flow than in postmenopausal women, indicating that the loss of estrogen diminishes β‐AR–mediated vasodilatory capacity (Guyenet 2006). Estrogen also modulates β‐AR expression in the heart. Following ischemia–reperfusion (I/R) injury, OVX increased β_1_‐AR expression and decreased β_2_‐AR expression, both of which were restored by estrogen supplementation, with β_2_‐AR levels even surpassing those in sham‐operated Sprague–Dawley rats (Wu et al. 2008). Moreover, in female rat hearts, both β_1_‐ and β_2_‐AR mRNA and protein levels were upregulated after arteriovenous fistula (AV‐shunt) creation, a response attenuated by 17β‐estradiol treatment (Dent et al. 2012).

**FIGURE 5:**
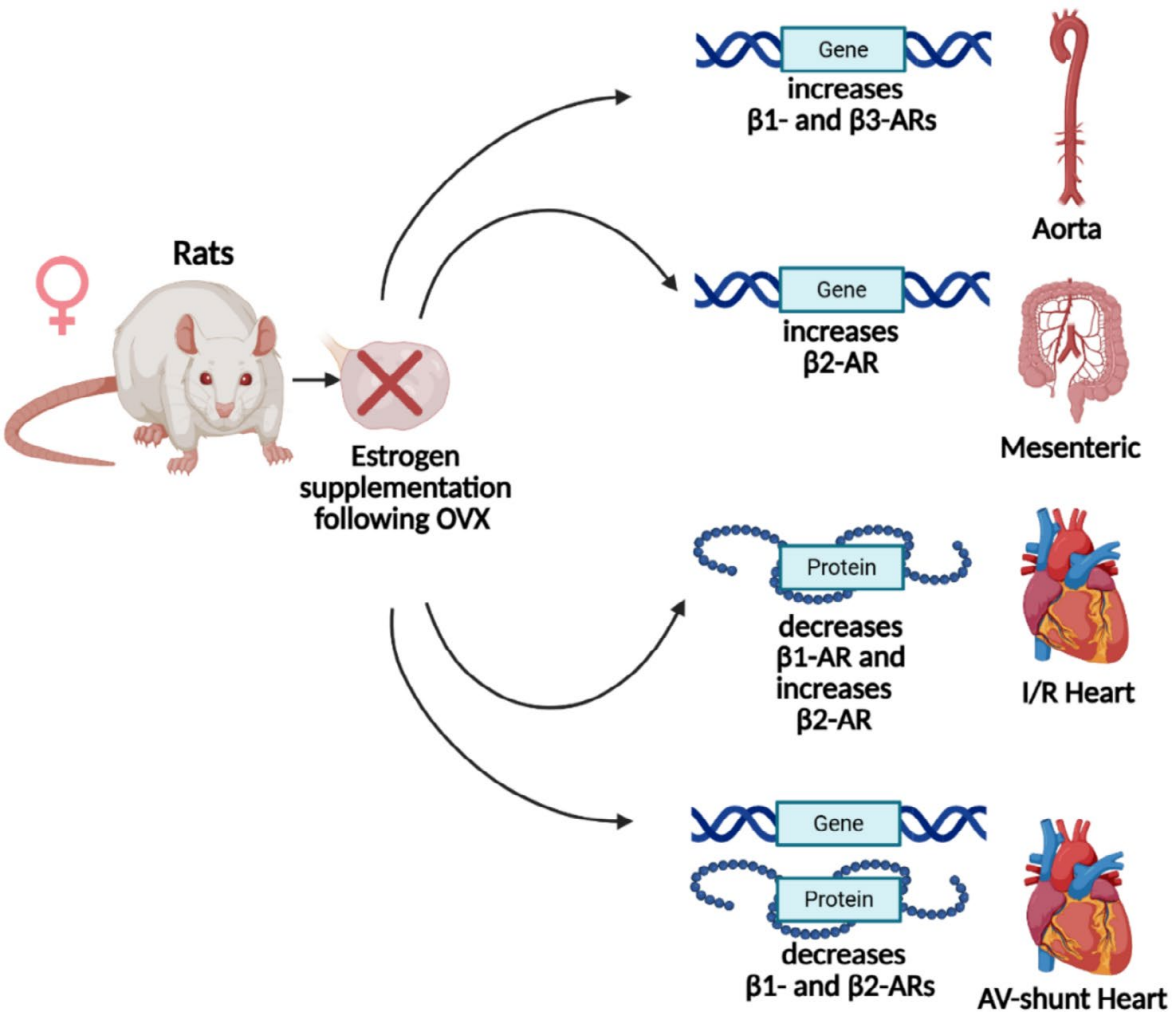
Estrogen modulation of β‐ARs expression in the cardiovascular system. Schematic summary of the tissue‐specific effects of estrogen on β‐ARs gene and protein expression in female rats. Created with BioRender.com.

In non‐cardiovascular tissues, estrogen's effects on β_3_‐AR are variable. Some studies report increased β_3_‐AR density in murine adipocytes in response to estrogen treatment, whereas others observe decreased β_3_‐AR expression in brown adipose tissue of female rats compared to control (Malo and Puerta 2001; Monjo et al. 2005). Nonetheless, estrogen treatment enhances β_3_‐AR–mediated relaxation in detrusor smooth muscle of female rabbits compared to the untreated group (Yono et al. 2000). A schematic summary of these findings is presented in Figure 5.

### 2.5 β‐Adrenergic–Estrogen Interactions in Vascular Aging: Implications for the Timing Hypothesis

Research demonstrates clear sex‐specific differences in HTN prevalence. Men are more susceptible before age 60, whereas women exhibit higher risk post‐menopause, likely due to declining estrogen levels (Aryan et al. 2020). The Framingham Heart Study reported a twofold increase in cardiovascular events among postmenopausal compared with premenopausal women, highlighting estrogen's protective role (Liccardo et al. 2022). Postmenopausal HTN increases stroke risk and contributes to higher prevalence of IHD, often through non‐obstructive mechanisms such as coronary microvascular dysfunction and altered vascular tone (Garcia et al. 2016; Robison et al. 2019; DeFilippis and Van Spall 2021). Women who experience premature menopause or primary ovarian insufficiency are at increased risk for coronary heart disease and CVD‐related mortality, further supporting the protective effects of endogenous estrogen (Garcia et al. 2016).

Estrogen exerts vascular protection primarily by modulating endothelial function. It enhances NO production via eNOS and regulates prostacyclin and thromboxane A2, both of which influence vascular tone (Novella et al. 2012). Endothelial vasodilator function declines rapidly during menopause, and reduced estrogen receptor (ER) expression or sensitivity may limit therapeutic efficacy in late postmenopausal women (Somani et al. 2019).

Despite its vasoprotective effects, estrogen therapy outcomes are context dependent. The Women's Health Initiative (WHI) and Heart Estrogen Progestin Replacement Study (HERS) reported variable cardiovascular responses, with postmenopausal HRT occasionally increasing risks of stroke, myocardial infarction, deep vein thrombosis, and pulmonary embolism (Xu et al. 2023).

The “timing hypothesis” suggests that estrogen confers vascular protection when therapy is initiated early in the menopausal transition but may lose efficacy or become detrimental if introduced after prolonged estrogen deficiency or established vascular dysfunction. The Early Versus Late Intervention Trial with Estradiol (ELITE) demonstrated that therapy timing significantly influences vascular outcomes (Garcia et al. 2016; SenthilKumar et al. 2023). Data from Hodis et al. (2012) support a “window‐of‐opportunity,” showing that HRT initiation before age 60 or within 10 years of menopause, continued for 6 years or more, maximizes reduction in coronary heart disease and overall mortality. Furthermore, estradiol slows carotid intima‐media thickness progression when therapy begins in early postmenopause but accelerates it if initiated later, consistent with the timing hypothesis (Sriprasert et al. 2019). Mechanistic studies indicate that estrogen protects the endothelium and slows plaque development in early atherosclerosis but may promote plaque rupture later (Santen 2017). Animal models support these findings. Early estrogen replacement post‐ovariectomy protects against ischemic stroke, whereas delayed therapy diminishes benefits and increases pro‐inflammatory responses, particularly in females (Liu et al. 2012; Robison et al. 2019).

Aging and vascular pathology are linked to reduced β‐AR responsiveness, mirroring the decreased effectiveness of estrogen when exposure is delayed or vascular dysfunction is already established (Schutzer and Mader 2003). In young women, β‐AR–mediated vasodilation counteracts sympathetic vasoconstriction, a response that diminishes with aging, resulting in unopposed α‐adrenergic vasoconstriction and higher blood pressure in older women (Baker et al. 2016). Aging further impairs β‐AR responsiveness downstream of the receptor via reduced Gs protein and AC activity (Abrass et al. 1982; Gaballa et al. 2000). Additionally, aging has been linked to a shift in β_3_‐AR signaling from Gs toward Gi dominance in ras, which may contribute to reduced receptor responsiveness in older individuals (López‐Canales et al. 2016). Endothelium removal nearly abolishes β‐AR–mediated vasodilation in older vessels, reflecting a high endothelial dependence with age (van der Zypp et al. 2000). Sex differences in α‐adrenergic vasoconstriction and β‐AR expression are high between ages 45–65 in women but decline with advancing age (Al‐Gburi et al. 2017b).

Collectively, these studies highlight the complex, context‐dependent regulation of β‐ARs by estrogen, which varies by species, sex, age, and tissue type. Observed differences among rats, mice, and human cells underscore the need for careful interpretation of preclinical data in cardiovascular research. These findings suggest that the physiological and translational relevance of estrogen–β‐AR interactions is highly context‐specific. Furthermore, estrogen's vascular effects are influenced by timing and endothelial health, emphasizing the importance of stage‐specific strategies to preserve cardiovascular function in women. To provide an integrated translational overview, the key mechanisms and species‐specific patterns are summarized in Table 2, highlighting both conserved and divergent features across experimental models and humans.

**TABLE 2:** Translational summary of β‐adrenergic and estrogen cardiovascular mechanis across mouse, rat, and human systems.

Mechanism/observation	Mouse	Rat	Human	Key references
**ER and β‐AR signaling**				
1. β_1_‐AR Gs coupling	+	+	+	Bristow et al. (1990) and Liccardo et al. (2022)
2. β_2_‐AR coupling (Gs/Gi)	+	+	+	Xiao et al. (1995), Xiao et al. (1999), Xiao (2001) and Liccardo et al. (2022)
3. β_3_‐AR in myocardium (Gi coupling)	NA	+	+	Gauthier et al. (1996), Gauthier et al. (1998), Zhang et al. (2005) and Cannavo and Koch (2017)
4. β_3_‐AR in myocardium (Gs/Gi coupling/isoform‐specific)	+ (β_3_a/β_3_b)	NA	NA	Hutchinson et al. (2002) and Sato et al. (2005)
5β_3_‐AR in vascular endothelium (Gi → NO)	+	+	+	Rozec and Gauthier (2006)
6. Prolonged β‐AR → enhanced NO	NA	+	NA	Davel et al. (2015)
7. Prolonged β‐AR → β_2_‐AR/Gi → ERK1/2 phosphorylation at Thr_202_/Tyr_204_ → eNOS uncoupling & oxidative stress; compensation nNOS/H_2_O_2_/caveolin‐1 → eNOS	+	NA	NA	Davel et al. (2014) and Bernak‐Oliveira et al. (2021)
8. COX inhibition enhances non–subtype‐specific β‐AR	NA	NA	+	Limberg et al. (2016) and Fujii et al. (2017)
**ER and β‐AR signaling cross talk**				
1. ERα modulation of Gαs/cAMP signaling (↑PKA activity)	NA	+	NA	Ding et al. (2009)
2. GPR30 → ↑ cAMP via Gαs	NA	+	+	Thomas et al. (2005) and Zucchetti et al. (2014)
3. GPR30 (GPER) → MAGUK/AKAP5 → ↓ AC → ↓ cAMP production; GPR30 (GPER) → AKAP5 → ↑ PDE → ↑ cAMP breakdown	NA	NA	+	Broselid et al. (2014) and Machuki et al. (2018)
4. Nebivolol → β_3_‐AR/ERβ/NO vasodilation	NA	+	NA	Garbán et al. (2004) and Feng et al. (2012)
5. Estrogen inhibition of catecholamine secretion (systemic SNS regulation). Estrogen restoration of catecholamine imbalance after gonadectomy. ER‐dependent modulation of β‐AR signaling.	NA	+	+	Sudhir et al. (1997), Sung et al. (1999), Gomes et al. (2012) and Riedel et al. (2019)
6. Localization of β‐ARs and ERs in caveolae (membrane microdomains)	+	+	+	Schlegel et al. (1999), Rybin et al. (2000) and Sato et al. (2012)
7. GPER agonist (G‐1) cardioprotection via β_1_−/β_2_‐AR interaction. G‐1 normalization of β_1_‐AR and upregulation of β_2_‐AR expression	NA	+	NA	Kang et al. (2012)
**Estrogen modulation of β‐AR receptor expression in the cardiovascular system**	NA	+	NA	Summarized in Figure 5
**Aging‐related changes on the cardiovascular system**				
1. Menopause‐related differences in CVDs	NA	NA	+	Garcia et al. (2016), Robison et al. (2019), Aryan et al. (2020), DeFilippis and Van Spall (2021) and Liccardo et al. (2022)
2. Variable cardiovascular effects of HRT (risk vs. benefit)	NA	NA	+	Xu et al. (2023)
3. Timing‐dependent effects of estrogen therapy	+	+	+	Hodis et al. (2012), Liu et al. (2012), Garcia et al. (2016), Santen (2017), Robison et al. (2019), Sriprasert et al. (2019) and SenthilKumar et al. (2023)
4. Aging‐related changes in endothelial function, and ER expression/sensitivity	NA	NA	+	Somani et al. (2019)
5. Aging‐related changes in adrenergic signaling	NA	+	+	Abrass et al. (1982), Baker et al. (2016), López‐Canales et al. (2016), Al‐Gburi et al. (2017b), Gaballa et al. (2000), van der Zypp et al. (2000) and Schutzer and Mader (2003)

*Note:* +, detectable; NA, not assessed. Detailed differences in β‐AR distribution across species, sex, vascular beds, and endothelial context are summarized in Table 1.

## 3 Conclusion and Future Prespectives

The distribution and function of β‐ARs, as well as their interactions with estrogen, are most likely influenced by tissue specificity, sex, age, species, strains and endothelium. Consequently, careful interpretation of experimental data is essential when translating findings into clinical contexts. Future studies should control these variables by initially using a single strain within a species, and systematically evaluating multiple vascular beds, including both conduit arteries and resistance vessels, within the same model. Conclusions should be limited to the specific strain, species, and vascular beds examined. Once reproducibility is confirmed within a model, findings can then be validated across additional strains, species, and ultimately human tissues to enhance translational relevance.

In addition, standardization of experimental variables such as physiological or pathological model, age, sex is necessary. Furthermore, careful selection and control of the type and concentration of preconstrictor agents are required to determine whether estrogen‐induced relaxation depends on the level or mechanism of preconstrictionchoice and concentration of preconstrictor agents is necessary to determine whether estrogen‐induced relaxation depends on the level or mechanism of preconstriction. Variability in vascular responses to estrogen across studies may also arise from fluctuations in endogenous estrogen levels in females. Therefore, it is important to examine whether normal physiological fluctuations, as well as hormonal status (e.g., intact versus ovariectomized animals), influence vascular reactivity. Consistent control of these conditions across studies and species will be critical for resolving discrepancies and improving reproducibility.

Finally, studies utilizing genetic knockout models targeting β‐ARs and ERs will be invaluable in defining receptor‐ and pathway‐specific mechanisms underlying the regulation of vascular tone. Genetic knockout models offer a powerful approach to dissect the specific roles of individual β‐AR subtypes, providing mechanistic insights beyond those achievable with pharmacological interventions alone. However, mouse models in general exhibit species‐specific differences in vascular and cardiac β‐AR signaling and in estrogenic modulation, which may limit their translational relevance to humans. In contrast, rats display more pronounced sex‐specific vascular responses to estrogen, suggesting that they may serve as a more appropriate model for studying β‐AR–mediated estrogenic effects. The development and use of genetic knockout models in rats therefore represent a promising strategy to bridge the gap between mechanistic studies and human physiology. Complementary studies in human vascular cells are essential to validate and refine these findings, further enhancing their clinical relevance. This integrative approach is essential to delineate mechanisms with translational relevance to human physiology while accounting for the inherent limitations of each experimental model.

## Author Contributions

B.E. conceived and designed the manuscript, performed the literature review, drafted the original manuscript, prepared the figures, and critically revised and approved the final version. I.K. contributed to manuscript revision. B.Z. generated the experimental data used in Figure 3. A.S. and S.S. assisted with manuscript revision. A.D. provided conceptual input and critically revised the manuscript. All authors read and approved the final version of the manuscript.

## Funding

The research was supported by institutional funding from the Institute of Physiology at TU Dresden. Basant Elsaid gratefully acknowledges financial support for living expenses during her research from the German Egyptian Research Long‐Term Scholarship Program (DAAD‐GERLS), the Cultural Affairs and Missions Sector in Egypt, the Association of Friends and Sponsors of TU Dresden (GFF), and the TU Dresden Graduate Academy (Completion and Wrap‐up Grant).

## Ethics Statement

All animal procedures were conducted in accordance with institutional guidelines and approved by the Institutional Ethics Committee of Technische Universität Dresden and the Saxony Regional Directorate (Landesdirektion Sachsen) under approval number TV T18/2023.

## Consent

The authors have nothing to report.

## Conflicts of Interest

The authors declare no conflicts of interest.

## Figures and Tables

**FIGURE A1:**
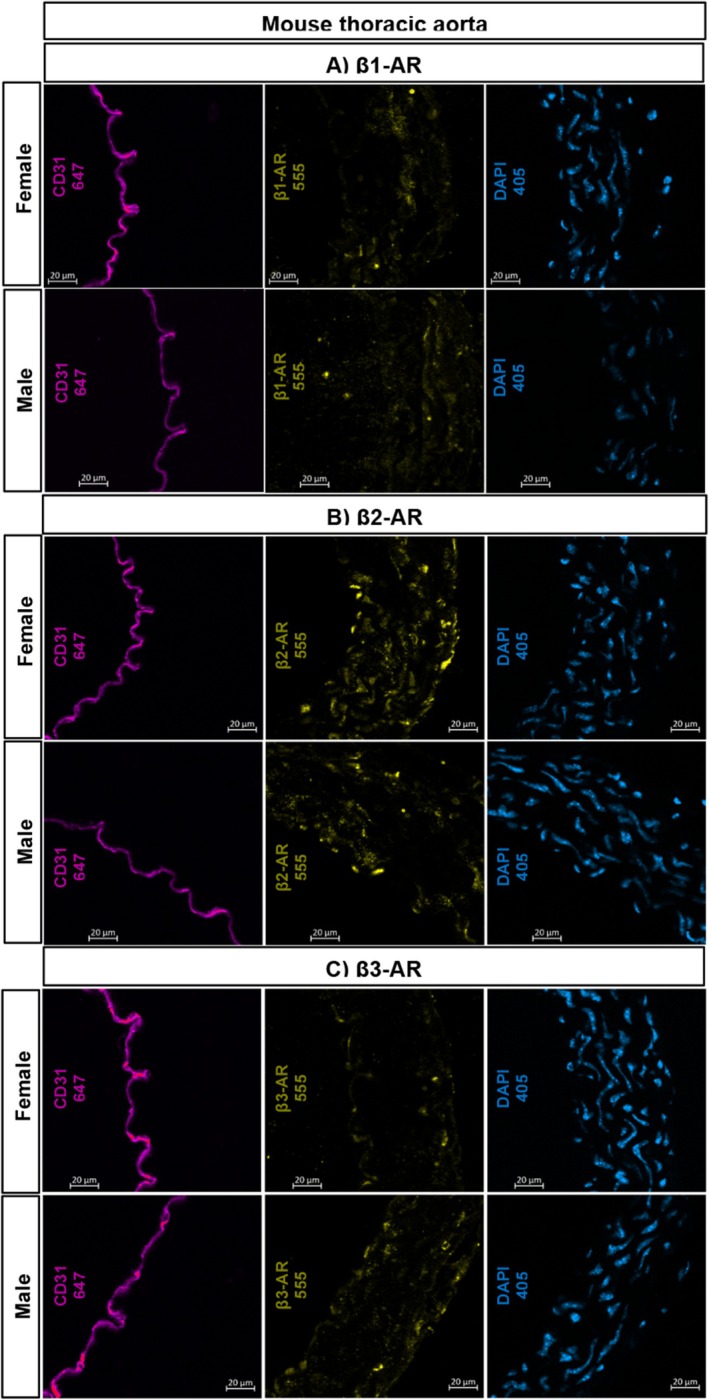
Distribution of β‐ARs in mice thoracic aorta. Representative fluorescence images of thoracic aorta sections from female and male mice. Endothelial cells were labeled using a primary anti‐CD31 antibody followed by an Alexa Fluor 647‐conjugated secondary antibody (magenta). Nuclei were counterstained with DAPI (blue). β‐ARs were detected using specific primary antibodies and Alexa Fluor 555‐conjugated secondary antibodies (yellow): (A) β1‐AR, (B) β2‐AR, and (C) β3‐AR.

**FIGURE A2:**
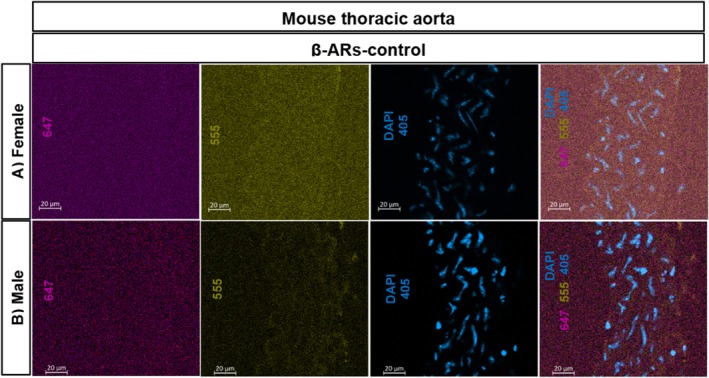
Immunofluorescence controls for β‐AR staining in male and female mouse thoracic aorta. Figures (A) and (B) show merged and individual fluorophore channel images of thoracic aortas from female and male mice, respectively. Samples were stained with donkey anti‐goat Alexa Fluor 647 (magenta), donkey anti‐rabbit Alexa Fluor 555 (yellow), and DAPI for nuclear staining (blue).

## Data Availability

The datasets generated during and/or analyzed in the current study are available from the corresponding author on reasonable request.

## Appendix A

### Isolation and Preparation of Mice Thoracic Aorta

All animal procedures were approved by the Institutional Ethics Committee of Technische Universität Dresden and Landesdirektion Sachsen (approval number TV T18/2023). Male and female C57BL/6J OlaHsd mice (16–18 weeks) were housed under standard conditions with free access to food and water. Euthanasia and isolation of mice aorta were performed as described by Elsaid et al. (2025).

### Mulvany Pin Myograph

Thoracic aortas were isolated from six male and six female mice. Experiments were conducted as described by Elsaid et al. (2025). Following pre‐constriction with phenylephrine (PE; 10^−6^ M; Sigma‐Aldrich, P6126), cumulative concentration‐response curves were generated using the β1/β2‐AR agonist isoprenaline (10^−11^–10^−6^ M; Tocris Bioscience, 1747) or the β3‐AR agonist BRL 37344 sodium salt (10^−9^–10^−4^ M; Tocris Bioscience, 0948) according to Al‐Gburi et al. (2017a).

### Immunofluorescence Staining

Immunofluorescence staining was performed following the detailed protocol described by Elsaid et al. (2025). Primary antibody incubation was carried out overnight at 4°C using the following antibodies: rabbit polyclonal anti‐β1‐AR (1:100; Thermo Fisher Scientific, PA1‐049), rabbit polyclonal anti‐β2‐AR (1:200; Thermo Fisher Scientific, BS‐21452R), rabbit polyclonal anti‐β3‐AR (1:200; Thermo Fisher Scientific, PA5‐143874), and goat anti‐CD31/PECAM‐1 (1:150; R&D Systems, AF3628). Negative controls were treated with antibody diluent only.

Following primary incubation, slides were washed and incubated for 1 h at room temperature with secondary antibodies diluted 1:350 in DAPI solution (Roche, 10236276001; pre‐diluted 1:10,000 in PBS): donkey anti‐goat Alexa Fluor 647 (Invitrogen, A21447) and donkey anti‐rabbit Alexa Fluor 555 (Invitrogen, A31572).
